# Modulation of NKG2D Expression in Human CD8^+^ T Cells Corresponding with Tuberculosis Drug Cure

**DOI:** 10.1371/journal.pone.0070063

**Published:** 2013-07-26

**Authors:** Syeda S. Hassan, Jang-Eun Cho, Muhammad Akram, Katherine L. Fielding, Hazel M. Dockrell, Jacqueline M. Cliff

**Affiliations:** 1 Department of Immunology and Infection, London School of Hygiene & Tropical Medicine, London, United Kingdom; 2 Department of Infectious Disease Epidemiology, London School of Hygiene & Tropical Medicine, London, United Kingdom; 3 Gulab Devi Chest Hospital, Lahore, Punjab, Pakistan; National Institute of Infectious Diseases, Japan

## Abstract

**Background:**

Biomarkers predicting tuberculosis treatment response and cure would facilitate drug development. This study investigated expression patterns of the co-stimulation molecule NKG2D in human tuberculosis and treatment to determine its potential usefulness as a host biomarker of tuberculosis drug efficacy.

**Methods:**

Tuberculosis patients (n = 26) were recruited in Lahore, Pakistan, at diagnosis and followed up during treatment. Household contacts (n = 24) were also recruited. NKG2D expression was measured by qRT-PCR in RNA samples both e*x vivo* and following overnight mycobacterial stimulation *in vitro.* Protein expression of NKG2D and granzyme B was measured by flow cytometry.

**Results:**

NKG2D expression in newly diagnosed tuberculosis patients was similar to household contacts in *ex vivo* RNA, but was higher following *in vitro* stimulation. The NKG2D expression was dramatically reduced by intensive phase chemotherapy, in both *ex vivo* blood RNA and CD8^+^ T cell protein expression, but then reverted to higher levels after the continuation phase in successfully treated patients.

**Conclusion:**

The changes in NKG2D expression through successful treatment reflect modulation of the peripheral cytotoxic T cell response. This likely reflects firstly *in vivo* stimulation by live *Mycobacterium tuberculosis,* followed by the response to dead bacilli, antigen-release and finally immunopathology resolution. Such changes in host peripheral gene expression, alongside clinical and microbiological indices, could be developed into a biosignature of tuberculosis drug-induced cure to be used in future clinical trials.

## Introduction

Tuberculosis (TB) remains a major pandemic health problem. In 2011, 8.7 million new cases were reported worldwide, of which 1.1 million cases were seen in HIV positive individuals, and 1.4 million people died [1]. Co-infection with HIV/AIDS and the emergence of drug resistant strains have made improved TB control an urgent necessity. Most immunocompetent people mount an effective immune response against the aetiological agent *Mycobacterium tuberculosis*, such that infection is controlled in a latent asymptomatic form in 90% of individuals [2].

TB treatment is long, leading to lack of adherence, acquired drug resistance and a 5% risk of relapse [3], [4]. There is no way of knowing if lung sterilization is achieved at an earlier time point; therefore biomarkers capable of predicting treatment efficacy/outcome are needed to aid clinical management [5], [6]. Biomarkers that can accelerate the drug developmental pipeline are also urgently needed, as current clinical trials use TB-relapse in a two year follow-up period to measure drug efficacy [7], although new recommendations suggest a shorter period would be sufficient [8].

CD8^+^ T cells play a pivotal role in TB immunity, particularly at later stages of infection and when bacillary burden is high [9]–[11]. There is a functional overlap between CD4^+^ and CD8^+^ T cells: both produce cytokines such as TNF-α and IFN-γ to activate *M. tuberculosis* infected dendritic cells and macrophages, however CD8^+^ T cells exhibit strong cyolytic activity enabling them to kill infected phagocytic cells: this activity has been observed in both mouse [12]–[14] and human [15]–[17] tuberculosis.

The NKG2D receptor is a member of the type II C-type lectin-like family of transmembrane proteins, and plays an essential role in both innate and adaptive immunity [18]. NKG2D is expressed as a homodimer on NK cells, γδ T cells, some CD4^+^ and CD8^+^ T cells [19]. NKG2D lacks signaling domains on its short intracellular tail and uses an adaptor molecule, DNAX-Activation Protein 10 (DAP10), to relay signals inside cells [19]. NKG2D binds a wide range of ligands that differ considerably in sequence, domain structure and affinity for the receptor [20] [21]. In humans, these ligands can be broadly divided into two families: major histocompatibility complex class I chain-related (MIC) and UL-16 binding proteins (ULBPS). The former family comprises highly polymorphic MICA and MICB, the expression of which are significantly upregulated on stressed, malignant and infected cells [22], including infection with *M. tuberculosis* [23].

In NK cells, NKG2D is a “master-switch” of activation that cannot be overcome by inhibitory signals. However, in T cells, NKG2D acts as a co-stimulatory receptor until a high activation status is reached, influenced by cytokine action, that enables it to act as an activation receptor and bypassing the conventional TCR-CD28 stimulation pathway [19]. Silencing of NKG2D and its adaptor molecules DAP10 and 12 by RNA interference leads to reduced cytotoxic activity of human NK and CD8^+^ T cells [24]. CD28 and NKG2D have overlapping but not identical functions. In mice, CD28 enhances CD8^+^ T cell function and survival whereas NKG2D only does the former [25]. In a tissue specific context, NKG2D expression is more important on antigen-specific lung resident effector T cells compared to memory ones [26]. The majority of studies have shown that NKG2D can act as a co-stimulatory molecule in its own right [27], [28], however Ehrlich *et al* found the opposite result i.e. NKG2D requires other co-factors such as CD28 to trigger CD8^+^ T cell proliferation, cytokine production and cytotoxicity in both murine and human cells [29].

NKG2D expression regulation is multi-layered; availability of DAP10, differential regulation by cytokines and chronic exposure to soluble or membrane bound NKG2D ligands control this pathway. This regulation may be awry in TB, due to its chronicity and the aetiological agent, *M.tuberculosis,* which has devised numerous immune evasion strategies. Some studies have shown that NKG2D may play a role in TB. Blocking NKG2D leads to inhibition of *M. tuberculosis-*specific CD8^+^ T cells *in vitro* [30] while NKG2D expression is up-regulated by mycobacterial stimulation *in vitro* [31]. Expression patterns of NKG2D have not been investigated in latent *M. tuberculosis* infection, active TB disease or during TB treatment in humans, despite its up-regulation upon mycobacterial stimulation *in vitro*.

The aim of this study was to determine the potential use of NKG2D as a host biomarker capable of predicting treatment response in human TB: this was achieved by measuring NKG2D mRNA and protein expression in healthy donors in the United Kingdom and in household contacts and newly diagnosed TB patients recruited in Lahore, Pakistan. We hypothesised that as NKG2D is involved in killing *M. tuberculosis-*infected cells, it would be more highly expressed in TB patients at diagnosis compared with household contacts, with a subsequent reduction in expression during successful TB treatment.

## Materials and Methods

### Ethics Statement

The study was approved by the LSHTM (study number 5459) and Gulab Devi Hospital Ethics committees. Informed written consent was obtained from all subjects for taking blood samples, analysis of mycobacterial immune responses and sample storage. The study was conducted according to the Declaration of Helsinki principles.

### Subject Recruitment and Venous Blood Sample Collection

In preliminary experiments, blood samples from healthy BCG-vaccinated donors were obtained from staff and students recruited at the London School of Hygiene & Tropical Medicine (LSHTM), U.K. Donors were BCG-vaccinated in adolescence, with blood samples collected more than ten years post-vaccination.

Subsequently twenty-six first-episode pulmonary tuberculosis inpatients were recruited at diagnosis into a longitudinal study, conducted at Gulab Devi Chest Hospital, Lahore, Pakistan in 2009/2010, and were treated according to prevailing Pakistan National Tuberculosis Program guidelines (2HRZE/6HE). Venous blood samples (10–30 ml) were obtained at diagnosis prior to treatment initiation, at the end of the two-month intensive phase and after completion of treatment (eight months). The inclusion criteria for patients included an age limit of 20–70 years, being HIV-negative and non-diabetic, haemoglobin level of ≥8g/dL and sputum smear positive and culture positive by solid culture on LJ slopes. Treatment outcomes were ascertained from patient records. Where possible, for each TB patient, blood samples were obtained from one adult household contact, defined as living in the same room when the TB patient was diagnosed (Table 1). QuantiFERON®-TB Gold (Cellestis, Ltd, Carnegie, Australia) was used in conjunction with lack of clinical symptoms or radiological evidence of TB to categorise the healthy household contacts as latently infected or not infected with *M. tuberculosis*. All the household contacts remained healthy and did not develop TB during a 1-year follow-up period after recruitment. Comparisons between TB patients and control subjects were with these household contact groups. For each patient or contact sample, 3 ml blood was collected into Tempus RNA tubes (Applied Biosystems) and stabilised by shaking vigorously for 10 seconds prior to freezing at−80°C. PBMC were isolated and stimulated immediately as described below, or frozen for downstream flow cytometry using cryoprotective reagent consisting of 10% DMSO (Sigma-Aldrich, Dorset, U.K.) in human AB serum-supplemented growth medium.

**Table 1:** Characteristics of tuberculosis patients and household contacts.

	TB patients (n = 26)	Household contacts (n = 24)
Age, years: median [range]	38 [20–60]	40 [20–65]
Gender: n, (% ) female	5 (20%)	15 (62%)
Classification of TB on CXR:	Moderate = 23, Mild = 3	N/A
Treatment Outcome	Cured^1^ = 24, Died = 2	
Study follow-up sample provided^2^	2months = 18, 8months = 6	N/A
Relationship to TB patient	N/A	Spouse = 5 Parent = 10 Child = 2 Sibling = 7
Infection status of household contact: n (%)	N/A	Latently infected^3^ = 13 (54%), Uninfected^4^ = 11 (46%)

1 Cured defined as sputum smear and culture negative at 8 months, with no clinical symptoms.

2 The loss-to-follow-up at 2 months was due to early hospital discharge and at 8 months was due to severe flooding in Pakistan in 2010.

3 Latently infected defined as IGRA-positive, normal chest X-ray and sputum smear negative.

4 Not infected defined as IGRA-negative, normal chest X-ray and sputum smear negative. N/A not applicable.

### Cell Isolation and Stimulation

Peripheral Blood Mononuclear Cells (PBMC) were separated from whole blood using density centrifugation over Ficoll-Histopaque 1077 (Sigma-Aldrich, Dorset, U.K.). For stimulation with live *M. tuberculosis*, PBMC from healthy BCG-vaccinated donors were cultured in L-glutamine supplemented RPMI-1640 (Invitrogen, U.K.) at 1x10^6^/ ml with *M. tuberculosis* H37Rv at a multiplicity of infection (MOI) of 1∶1 (bacilli: monocyte) for 7 days. CD4^+^ and CD8^+^ T cells were isolated from the PBMC cultures using Dynabeads (Dynal Biotech, Wirral, U.K.) and lysed and frozen at−80^°^C in RNAzol B (Biogenesis, Dorset, U.K.). Ten-fold diluted whole blood from BCG-vaccinated donors was incubated in supplemented RPMI-1640 with live *M. bovis* BCG or *M. tuberculosis* H37Rv at 1∶1 MOI, assuming peripheral blood contains 4x10^5^ monocytes/ml, for 7 days and the cell layer was lysed and frozen at−80^°^C in mRNA Stabilization reagent (Roche Applied Science). PBMC from TB patients and household contacts at 2x10^6^/ ml were cultured alone or stimulated for 16 hours with live *M. bovis* BCG at 10∶1 or 1∶1 MOI or immobilized 2 µg/ml anti-CD3 (Mabtech, Stockholm, Sweden) in supplemented RPMI-1640. Cell pellets were lysed and frozen at−80^°^C in RLT buffer (RNeasy kit; Qiagen, West Sussex, U.K.).

### RNA Extraction and qRT-PCR

RNA was extracted using the RNAzol protocol, the Roche mRNA isolation kit, Qiagen RNeasy extraction kit or the Tempus Spin extraction kit (Applied Biosystems) as appropriate for the sample, following manufacturers’ instructions. Any potentially contaminating DNA was removed by DNAse treatment, using on-column DNAse (Qiagen) where possible or alternatively Ambion *TURBO* DNA-*free*™ (Invitrogen). RNA quality was checked using a 2100 Bioanalyzer (Agilent Technologies) and quantified using the Ribogreen assay (Invitrogen).

Equivalent amounts of mRNA from each sample were reverse transcribed using oligo-dT primers and Superscript III (Invitrogen) in 20 µl reactions, and the reaction mix was then diluted to 100 µl prior to use. Quantitative RT-PCR (qRT-PCR) was performed with 5 µl cDNA using either an ABI Prism 7000 or 7500 Fast machine, and Applied Biosystems SYBRGreen reagents. Primer sequences were kindly provided by Dr Martin Holland for HPRT [32]. Other primers were designed using Primer3 software [33] and gene-specificity checked by nucleotide BLAST [34]: qRT-PCR primer sequence were NKG2D-Sense (S): CTGGTGAAGTCATATCATTGGATGG; NKG2D-Anti-sense (A): AGAATGGAGCCATCTTCCCACT; DAP10-S: CTGCCAGACCCCAGTCCAC; DAP10-A: TGGGAGCAAAAGCAGGAAGA; HPRT–S: GGCAGTATAATCCAAAGATGGTCA; HPRT–A: GTCTGGCTTATATCCAACACTTCGT; Cyclophilin A-S: GCTGGACCCAACACAAATGG; Cyclophilin A-A: TTGCCAAACACCACATGCTT.

### Flow Cytometry

Frozen PBMC were thawed by drop-wise addition of cold RPMI-1640 medium followed by centrifugation and two washes with RPMI-1640 medium prior to cell counting. The PBMCs were incubated overnight in growth medium at 37^°^C/5% CO_2_, with Brefeldin A (Becton Dickinson-BD) included for the last three hours. Single stain compensation tubes and fluorescence minus one (FMO) controls were used. One million PBMCs per tube were stained with anti-CD3 (PERCP-Cy 5.5, clone OKT3: e-Bioscience), anti-CD8 (FITC, clone OKT8:(e-Bioscience) and anti-NKG2D (PE, clone 1D11:BD) prior to adding fixation and permeabilisation solutions (BD). Intracellular staining was performed with a pre-titrated amount of anti-Granzyme B (Alexa-Fluor 647, clone GB-11: BD). Paraformaldehyde/PBS (10%) (Sigma) was added to the samples prior to running on a FACS Calibur 4CA E5180 (BD) under Category 3 conditions. Data were analysed using FlowJo (v7.6). Lymphocytes were gated using forward versus side scatter. Dead cells were excluded using Live/dead staining (Invitrogen).

### Data Analysis

All data were analysed using non-parametric tests in GraphPad Prism v5: the Wilcoxon signed rank test was used for paired data and the Wilcoxon rank sum test (Mann-Whitney test) for unpaired data. Expression changes within the successfully cured patient group were analysed separately from those who died, so that expression changes which might potentially correlate with treatment-response could be identified. Prior to patient recruitment, a sample size calculation based on pilot data indicated that a cohort of 23 patients and 23 contacts would have 90% power to detect a difference between TB patients and contacts of 2.9-fold (assuming standard deviation 3 in each group) with a type I error of 5%.

## Results

### Expression of NKG2D, but not DAP10, mRNA is up-regulated by CD8^+^ T Cells Following Mycobacterial Stimulation

**Figure 1:**
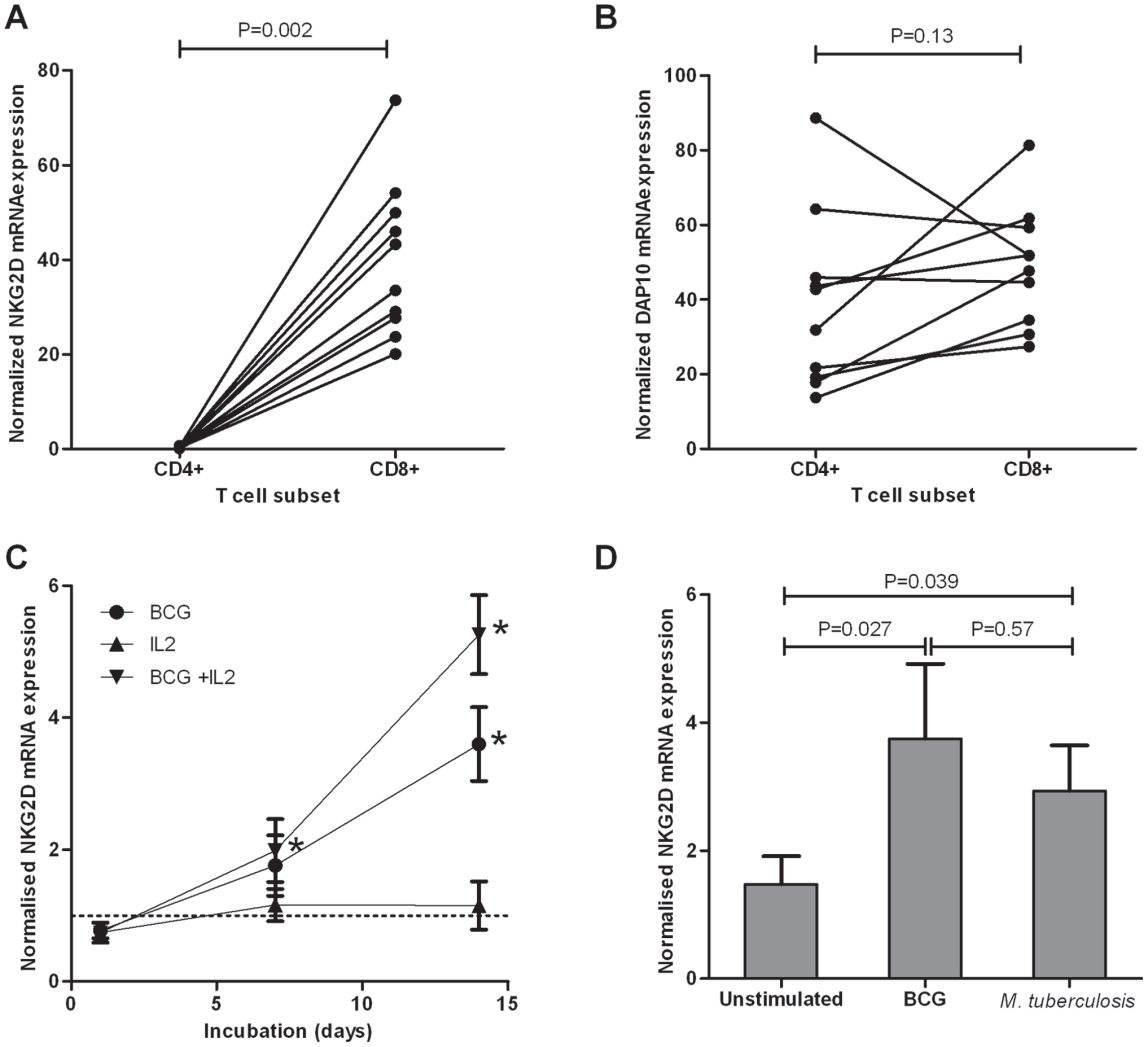
Regulation of NKG2D and DAP10 mRNA following mycobacterial stimulation *in vitro*. CD4^+^ and CD8^+^ T cells were isolated from ten healthy BCG-vaccinated donors after 7 days of PBMC stimulation with live *M. tuberculosis* H37Rv, on ten separate occasions. NKG2D (A) and DAP10 (B) mRNA expression levels in each T cell subset were determined by qRT-PCR. Data are normalised against the HPRT housekeeping gene, with the mean of duplicate technical replicates shown. (C) Diluted whole blood cultures from five healthy donors were incubated on five separate occasions with live *M. bovis* BCG in the absence or presence of IL-2 for the time indicated. NKG2D mRNA expression was determined for each sample in duplicate by qRT-PCR and is shown normalised against HPRT and normalised against the unstimulated control at each time point, with mean and standard error of the mean for the five donors shown. *P<0.05 compared to the medium control cultures. (D) Diluted whole blood cultures from nine BCG-vaccinated donors were incubated with live *M. bovis* BCG or live *M. tuberculosis* for six days in three separate experiments. NKG2D mRNA expression is shown normalised against HPRT mRNA expression, with the mean and standard error of the mean of the nine donors shown. Statistical comparisons were performed using the Wilcoxon signed rank test for paired data.

In a DNA-array study [31] we found that NKG2D mRNA expression was enhanced on CD8^+^ T cells compared to CD4^+^ T cells. Here, PBMC from ten BCG-vaccinated biologically independent donors were stimulated for 7 days with live *M. tuberculosis*, the CD4^+^ and CD8^+^ T cells were isolated and expression of NKG2D was assessed. NKG2D expression was highly elevated in CD8^+^ T cells (p = 0.002) (Figure. 1A). In contrast, there was no consistent expression pattern between CD4^+^ and CD8^+^ T cells for DAP10, which transduces NKG2D signals in CD8^+^ T cells (p = 0.13) (Figure. 1B). Thus NKG2D mRNA expression appears to be the limiting factor in NKG2D-DAP10 regulation in CD8^+^ T cells following *M. tuberculosis* stimulation. To determine whether NKG2D expression could be detected in whole blood, diluted whole blood cultures from five healthy individuals were incubated with live *M. bovis* BCG, and the level of NKG2D mRNA increased steadily throughout a 2-week incubation period (Figure 1C). This was enhanced by inclusion of IL-2, possibly by promoting cell survival or proliferation, or via a direct effect on NKG2D expression [19]. Similar levels of NKG2D expression were observed following stimulation with either *M. tuberculosis* or *M. bovis* BCG in diluted whole blood cultures (Figure 1D). These results showed that diluted whole blood cultures and *M. bovis* BCG stimulation could be a useful method for measuring changes in NKG2D expression in a more clinical setting.

### NKG2D *ex vivo* Blood Gene Expression is Unaltered During Active TB Disease but Changes with Successful Chemotherapy

**Figure 2:**
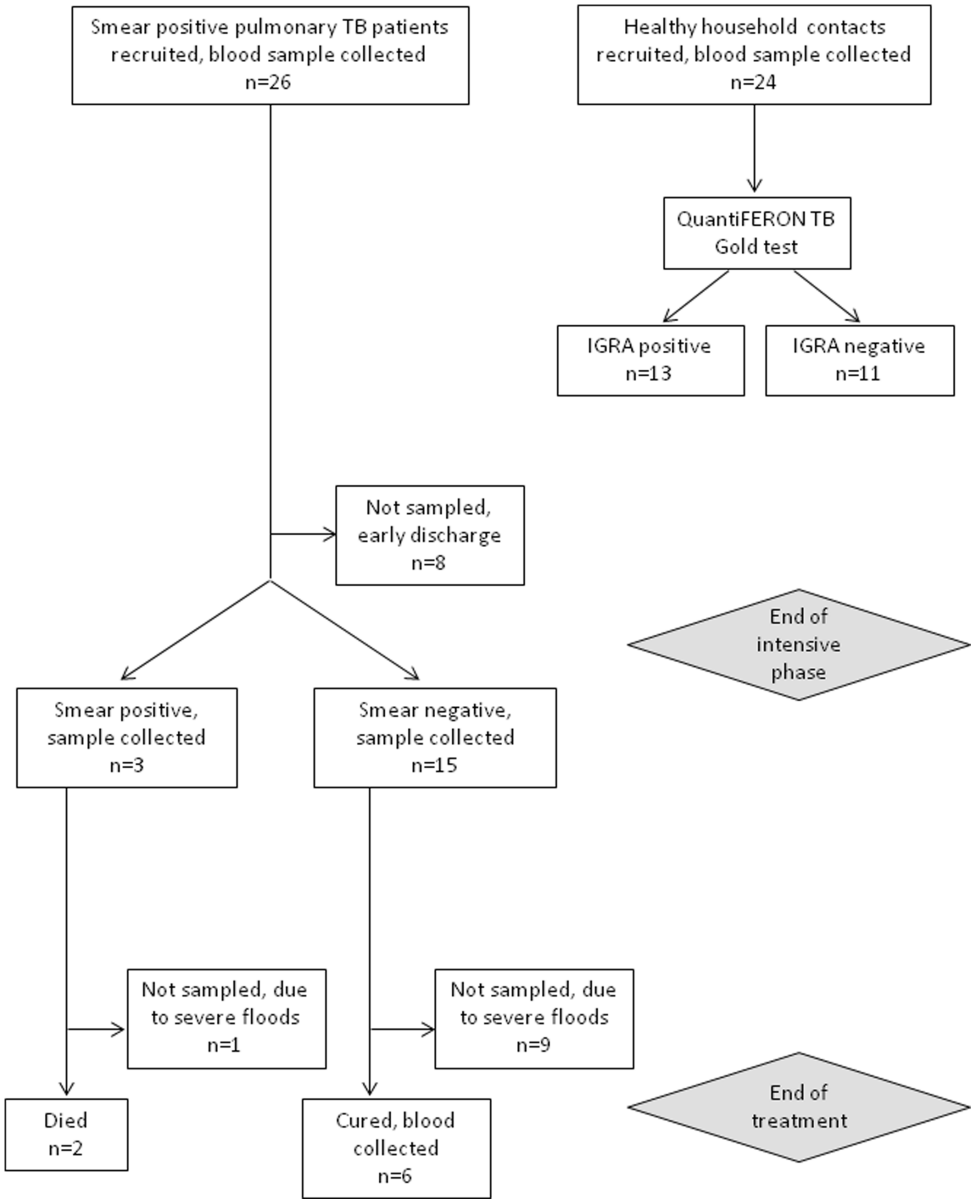
TB patient recruitment and follow-up. Sputum smear positive pulmonary TB patients were recruited in Lahore, Pakistan, from the inpatient department at Gulab Devi chest hospital. Blood samples were collected at diagnosis and where possible after the intensive treatment phase and after the full treatment phase: study follow-up success is shown. The number of patients who were sputum smear positive after the intensive phase is indicated. Apart from the two patients who died, all patients were sputum and culture negative at the end of treatment and classified as cured according to hospital records. Healthy household contacts of the TB patients were also recruited, and all had normal chest X-rays, no symptoms and were sputum smear negative.

As NKG2D was expressed by healthy donors’ CD8^+^ T cells following mycobacterial stimulation *in vitro,* we hypothesized that NKG2D gene expression would be elevated in active TB patients compared to contacts due to *in vivo* mycobacterial exposure. For this purpose, 26 pulmonary TB patients were recruited in Pakistan at the start of TB treatment, of whom 18 (69%) and 6 (23%) were followed up at 2 months and 8 months respectively (Table 1 and Figure 2). Twenty-four patients were cured at the end of treatment, all of whom remained disease free in the subsequent 12 month period. Two patients died during the continuation phase: these two patients remained sputum and culture positive after two months of treatment, and verbal autopsy indicated their likely cause of death was TB. At the same time as TB patient recruitment, 24 household contacts were recruited, of whom 13 (54%) were classified as latently infected and 11 (46%) as uninfected, based on their IFNγ responses in the QuantiFERON®-TB Gold assay.

**Figure 3:**
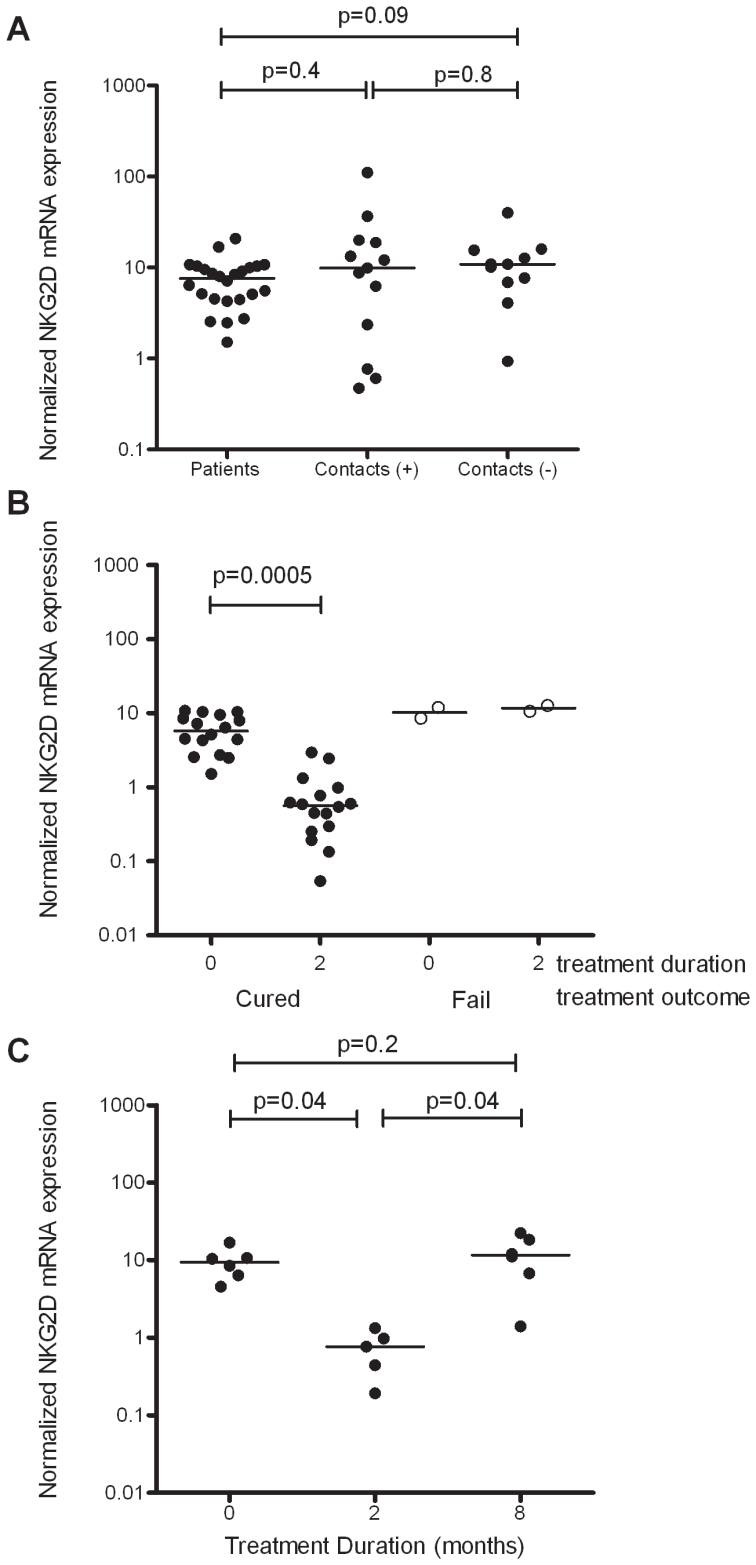
NKG2D *ex vivo* blood gene expression prior to and during chemotherapy. RNA was extracted from whole blood Tempus tubes and gene expression investigated by qRT-PCR. (A) NKG2D expression in *ex vivo* venous blood from untreated active TB cases at diagnosis (n = 26), in latently-infected household contacts (Contacts (+): n = 13) and in uninfected contacts (Contacts (−): n = 11), all recruited in Lahore, Pakistan. The Wilcoxon ranksum test was used to compare the clinical groups. (B) NKG2D expression after the end of the intensive phase in 16 patients who were eventually successfully cured (closed circles) and in 2 patients who subsequently died (open circles). (C) NKG2D expression during the entire course of TB chemotherapy in a different 6 patients, for whom data were available at the end of treatment. The Wilcoxon signrank test was used to analyse the paired data in (B) and (C). The lines in the centre of each group represent medians. NKG2D mRNA expression was determined by qRT-PCR, with results shown normalised against the housekeeping gene Cyclophilin A.

Contrary to expectation, there was no difference in NKG2D gene expression between untreated TB patients, latently infected and uninfected contacts at diagnosis, although a trend towards reduced expression in patients compared to uninfected contacts was observed (Figure 3A). There was considerable heterogeneity within the latently-infected group, possibly due to subjects possessing different degrees of subclinical infection. Next, we investigated the effect of anti-TB drug treatment on NKG2D expression. Among TB patients who were subsequently successfully cured, there was a decline in NKG2D mRNA expression from treatment initiation (median 5.8 arbitrary units) to the end of the 2-month intensive treatment phase (median 0.6 arbitrary units) (p = 0.0005) (Figure 3B). There was no apparent reduction in the NKG2D mRNA expression in the two patients who subsequently died (Figure 3B). In the six cured patients for whom data were also available at the end of TB treatment, this decline in NKG2D mRNA expression was observed in the intensive phase (p = 0.04), but was followed by an increase (p = 0.04) during the continuation phase (median at diagnosis 9.4, at 2 months 0.77 and at 8 months 11.6 arbitrary units) (Figure 3C).

### Active TB Patients Exhibit Enhanced NKG2D Expression Following Antigenic re-stimulation *in Vitro*

**Figure 4:**
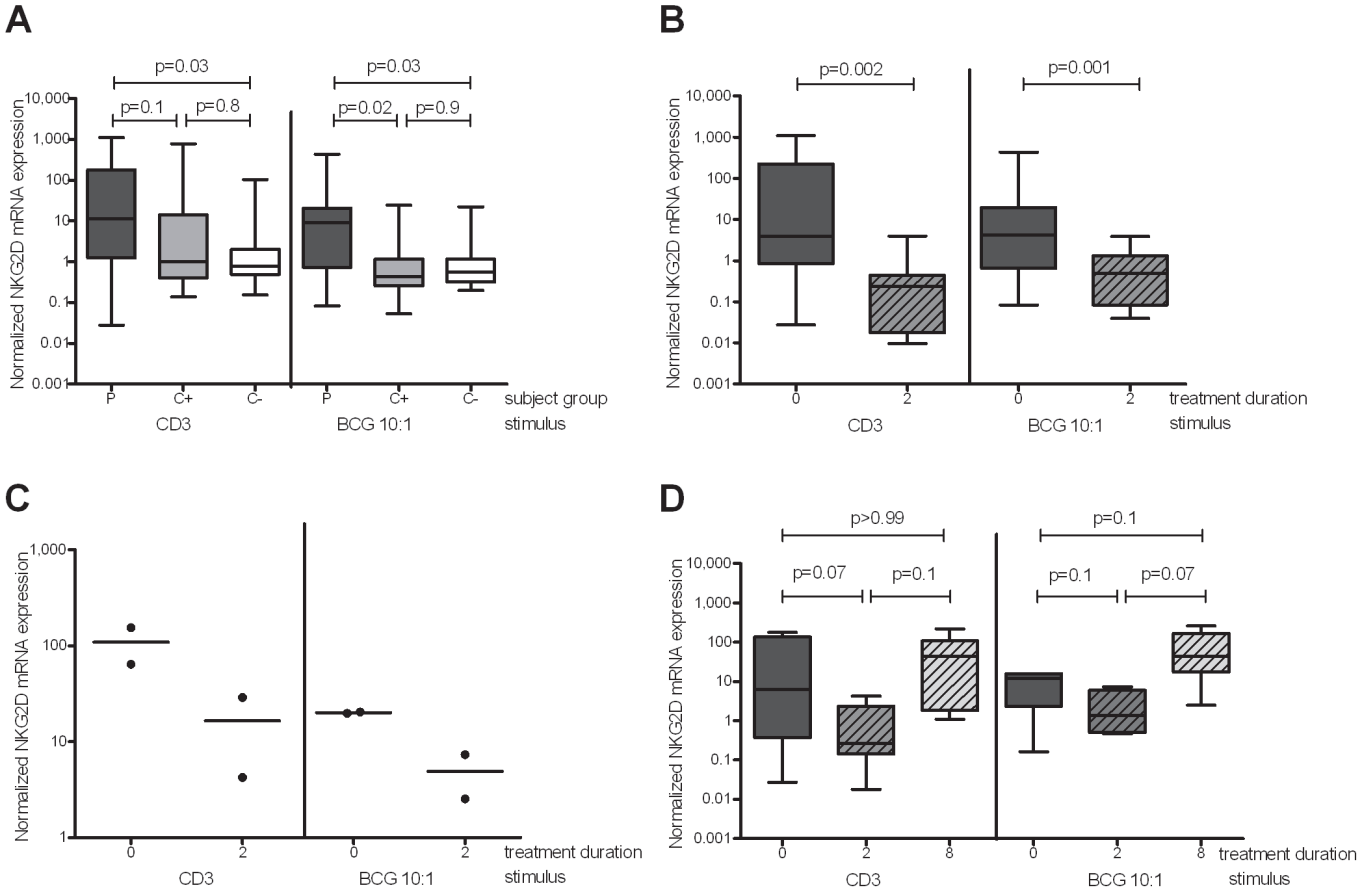
NKG2D gene expression at diagnosis and during chemotherapy in *in vitro* stimulated RNA samples. PBMC were isolated and stimulated *in vitro* with *M. bovis* BCG (MOI 1∶1) or anti-CD3 mAb for 16 hours and NKG2D mRNA expression analysed by qRT-PCR. (A) NKG2D mRNA expression at TB diagnosis in 26 untreated TB patients (P), 13 latently infected contacts (C+) and 11 uninfected contacts (C-). (B) Modulation of NKG2D mRNA expression during the intensive phase of treatment in 16 successfully cured TB patients. (C) NKG2D mRNA expression following stimulation in 2 patients who subsequently died. (D) NKG2D mRNA expression in a separate group of 6 successfully cured patients throughout the full treatment course. The line in the centre of the box and whisker plots represents the median whereas the top and bottom lines represent the 75^th^ and 25^th^ quartile respectively, and whiskers represent minimum and maximum data points. The Wilcoxon ranksum test (A.) and the Wilcoxon signrank test (B) and (D) were used for statistical analyses of unpaired and paired data respectively.

As the NKG2D expression in *ex vivo* blood was modified during active TB disease and TB chemotherapy, we investigated whether the NKG2D-mediated cytolytic potential upon antigenic re-stimulation was altered. PBMC from active TB patients expressed higher levels of NKG2D mRNA compared to both latently-infected (p = 0.02) and non-infected (p = 0.03) contacts in response to live *M. bovis* BCG stimulation (Figure 4A). Similar responses were seen for latently-infected and non-infected contacts (Figure 4A). A small difference between responses in active TB patients and uninfected contacts was also observed with anti-CD3 stimulation (p = 0.03) (Figure 4A). Among TB patients, we found a decline in antigen-induced NKG2D mRNA expression during the intensive phase of chemotherapy, for both *M. bovis* BCG (p = 0.002) and anti-CD3 stimulation (p = 0.001), in patients who were subsequently successfully cured (Figure 4B). There also appeared to be a reduction in the NKG2D expression following stimulation in the two patients who died, in contrast to the *ex vivo* expression levels (Figure. 4C). For both the *M. bovis* BCG and anti-CD3 stimulated samples, samples from the end of successful treatment showed a trend towards increased NKG2D expression (Figure. 4D).

### NKG2D Protein Expression is down-regulated by Intensive Phase TB Treatment

**Figure 5:**
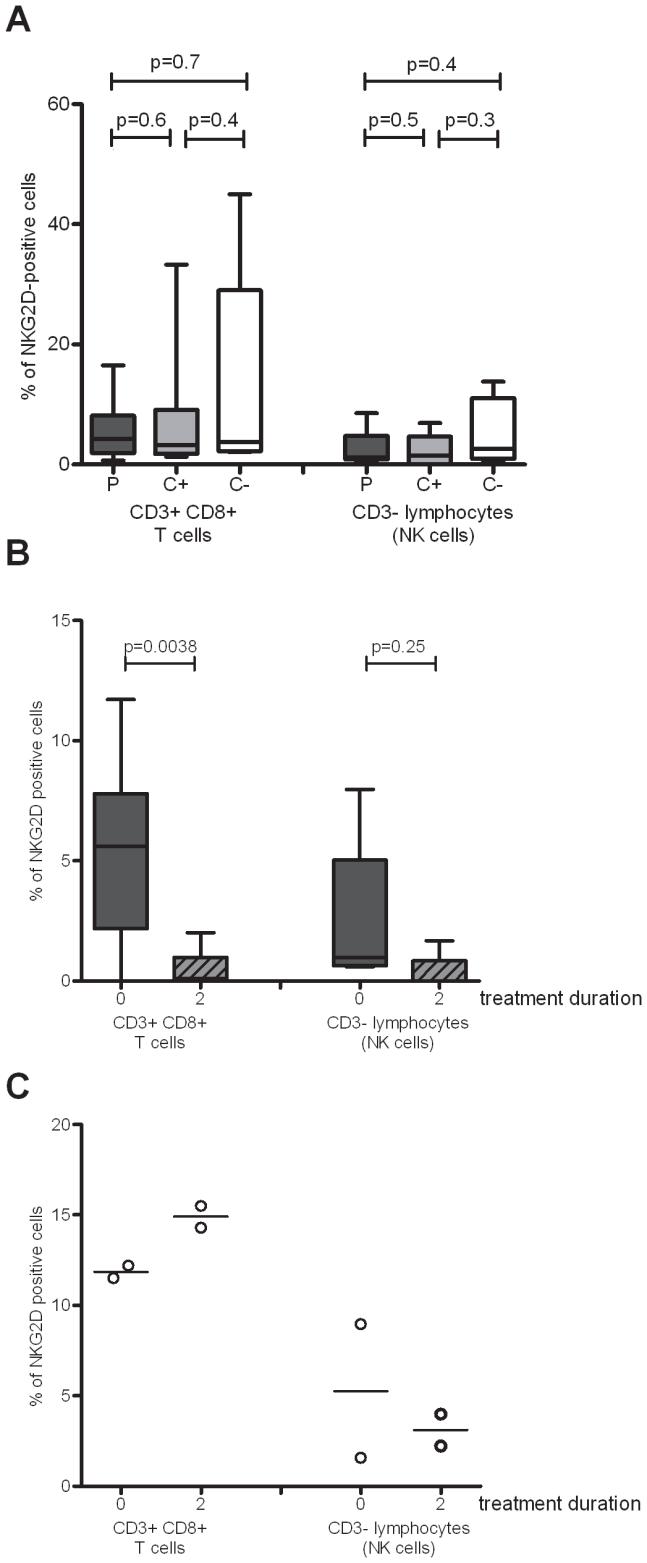
NKG2D protein expression during active TB disease and chemotherapy. Frozen PBMCs were thawed and stained to analyse NKG2D protein expression. (A) NKG2D protein expression on CD3^+^CD8^+^ T cells and CD3^-^ lymphocytes at diagnosis in 21 TB patients (P), 8 latently infected contacts (C+) and 6 uninfected contacts (C-). Data were analysed using the Wilcoxon rank sum test. (B) Modulation of NKG2D protein expression on CD3^+^CD8^+^ T cells and CD3^-^ lymphocytes with the intensive phase of chemotherapy in successfully cured patients (n = 13). The graphs are box and whisker plots, as described in Figure 3, and analysed using the Wilcoxon signed rank test. (C) NKG2D protein expression in 2 patients who subsequently died.

As NKG2D mRNA expression was modulated by TB chemotherapy, we next investigated whether these mRNA changes were translated into changes in protein expression. No differences in NKG2D protein expression were found between TB patients at diagnosis, latently infected contacts and uninfected contacts in the total lymphocyte gate (not shown), nor in the CD3^+^CD8^+^ cell subset or the CD3^-^ lymphocyte subset which would have contained NK cells (Figure 5A). The NKG2D protein expression was decreased during the first 2 months of successful TB treatment in the CD3^+^CD8^+^ T cell compartment (p = 0.0038), whereas the expression within the non-T cell lymphocyte compartment was more similar at diagnosis and 2 months (p = 0.25) (Figure 5B). In contrast, in the two patients who subsequently died, there was no apparent decrease in the NKG2D protein expression, in either the CD8^+^ T cell or the non-T cell compartments (Figure 5C). Thus the decline in NKG2D protein expression in the first two months of chemotherapy confirmed the mRNA expression results.

### NKG2D/Granzyme B Protein co-expression Increases with the Intensive Phase of Treatment

**Figure 6:**
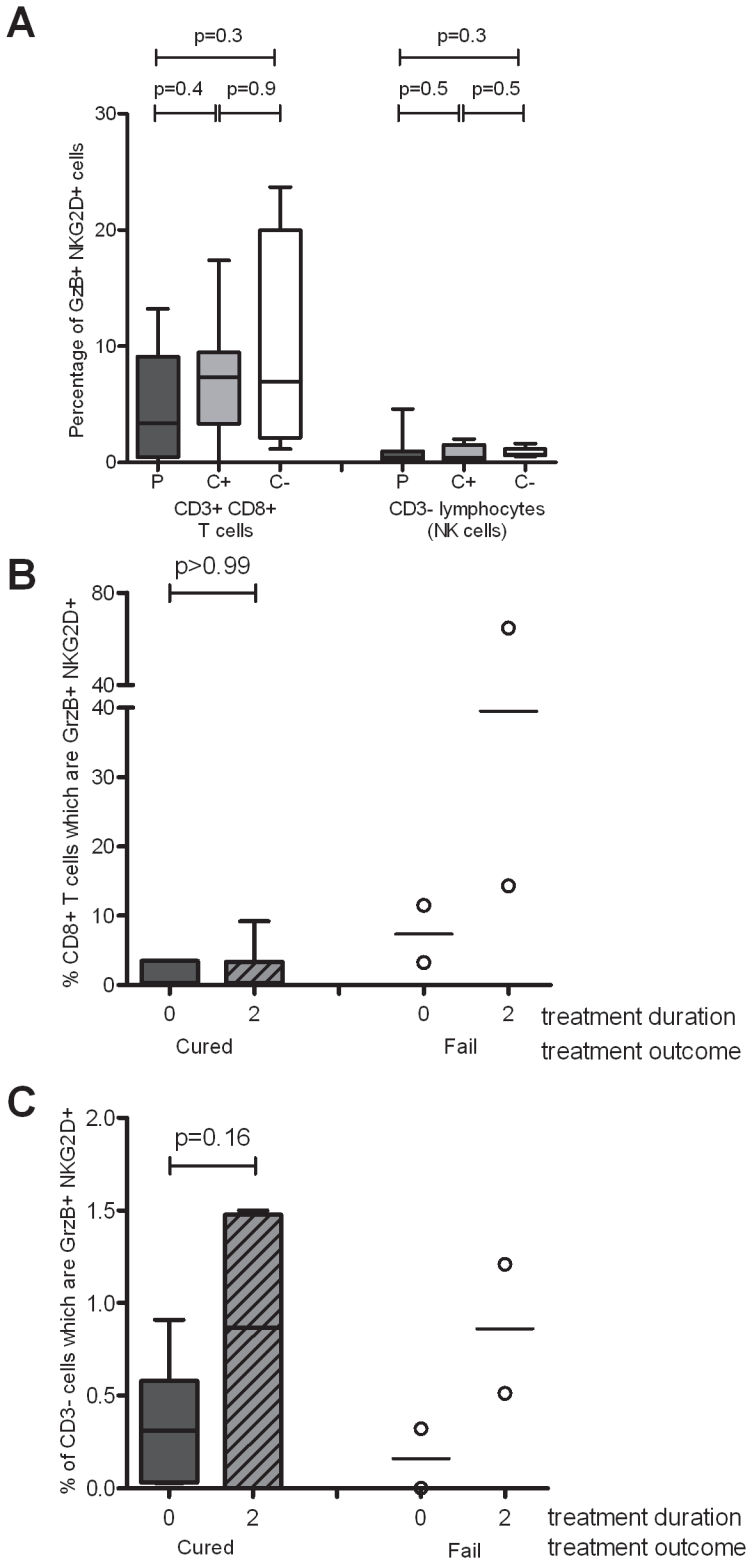
Modulation of NKG2D/GrzB protein co-expression during active TB disease and chemotherapy. Frozen PBMCs were thawed and NKG2D/GrzB co-expression was investigated by flow cytometry. (A) Percentage of NKG2D GrzB double-positive cells in the CD3+CD8+ lymphocyte gate or CD3- lymphocyte (NK cell) gate at diagnosis in TB patients (P: n = 18), latently infected contacts (C+: n = 7) and uninfected contacts (C-: n = 4). Modulation of NKG2D/GzB co-expression with the intensive phase of chemotherapy on CD3^+^CD8^+^ T cells (B) or CD3- lymphocytes (NK cells) (C) in patients who were successfully cured (n = 8) or who subsequently died (n = 2). The graphs are box and whisker plots, as described in Figure 3. The Wilcoxon ranksum test (A.) and the Wilcoxon signrank test (B) and (C) were used for statistical analyses of unpaired and paired data respectively.

We next investigated NKG2D expression in concert with Granzyme B expression, to establish whether NKG2D has an impact on cytotoxicity and degranulation in active TB and during chemotherapy. No differences were found between NKG2D/Granzyme B co-expression between TB patients at diagnosis, latently infected and uninfected contacts, in either the CD3^+^CD8^+^ cell subset or the CD3^-^ lymphocyte subset (Figure 6A). NKG2D/Granzyme B co-expression remained unchanged in CD8^+^ T cells after the intensive phase of treatment in successfully cured patients (p>0.99) (Figure 6B): the two patients who subsequently died did not fit with this pattern of consistent low level NKG2D/Granzyme B co-expression. NKG2D/Granzyme B co-expression in the non-T cell lymphocyte compartment appeared to be higher at the end of the intensive phase compared with at diagnosis, in both the cured patients and those who later died (Figure 6C), though statistical evidence was weak (p = 0.16 for cured patients).

## Discussion

We have demonstrated that NKG2D expression was regulated in a complex, biphasic manner during TB drug treatment in TB patients. To our knowledge, this is the first study that has investigated NKG2D gene and protein expression on CD8^+^ cytotoxic T cells during active TB disease, latent infection and TB treatment. Contrary to our expectation, the *ex vivo* NKG2D mRNA expression and lymphocyte protein expression in blood was not significantly different in active TB disease compared to latently-infected or non-infected household contacts at diagnosis. There was, however, a dramatic reduction in NKG2D expression within the 2 month intensive treatment phase, and this was followed by a return to high levels by the end of treatment in successfully cured patients. There was no modulation of NKG2D expression in two patients who died during the continuation phase.

NKG2D is expressed by cytotoxic cells, where it is involved in cytolytic killing [20], [35], and it can mediate costimulation for CD8^+^ T cells [25], promoting a Tc1 phenotype [36]. In this study, NKG2D mRNA was highly expressed in CD8^+^ T cells compared to CD4^+^ T cells in healthy donors following stimulation *in vitro* with mycobacteria. In contrast, DAP10 mRNA was expressed in both T cell subsets, indicating that expression of the two protein subunits of the receptor complex are regulated via different mechanisms, in accordance with a previous study [37]. The modulation of NKG2D expression in TB patients with successful treatment occurred in CD8^+^ T cells, and to a lesser extent on NK cells, as determined by flow cytometry. CD8^+^ T cells are crucially involved in protection against *M. tuberculosis*: patients with active TB disease have fewer circulating terminally-differentiated CD8^+^ T cells than latently-infected healthy subjects, with a reduction in the proportion of polyfunctional CD8^+^ T cells and lower perforin and IFNγ production [38].

The biphasic nature of the changes in NKG2D expression with treatment might reflect a combination of altered sequestering of lymphocytes in the lungs, varying with changes in bacterial burden and antigen level, together with altered differentiation stages of circulating lymphocytes and altered general ability to respond to antigenic stimulation. Overnight stimulation of diluted blood *in vitro*, either with live mycobacteria (*M. bovis* BCG) or anti-CD3 mAb, enhanced NKG2D expression in untreated TB patients, compared to that in uninfected controls or after two months of treatment. This could imply that there were more differentiated effector cells in the blood capable of responding rapidly to antigenic stimulation. Alternatively, the reduced NKG2D expression at month 2 of treatment might reflect a loss of a cytotoxic lymphocyte population, possibly due to cellular exhaustion, which is later replenished, coinciding with cure and immune restoration. Although it is possible that the antibiotics themselves had a direct effect on NKG2D expression, we consider this unlikely as in previous transcriptomic studies, we and other investigators have seen no evidence of a direct effect on immune cell gene expression profiles [39], [40]. Furthermore, if the change in gene expression was due to a non-specific toxic effect, one would also expect it occur in the two patients who died, but this was not the case. Future studies will be aimed at testing the direct effects of anti-mycobacterial treatment on peripheral gene expression, to formally rule out this possibility.

NKG2D has previously been shown to be upregulated on CD8^+^ T cells from healthy donors following stimulation with live *M. tuberculosis* [31] or *M. tuberculosis* extract [23]. One study examining NKG2D expression on NK cells in TB patients found no difference to that in controls [41], in agreement with the present study. The potential importance of NKG2D in controlling *M. tuberculosis* infection has been demonstrated in mouse models, whereby CD8^+^ T cell-mediated cytotoxicity is impaired in mice deficient for DAP10, the NKG2D signaling adapter molecule [42]. IL-15-deficient mice are highly susceptible to *M. tuberculosis*, and this defect is mediated in part by reduced NKG2D expression [30]. NKG2D expression is also modulated by IL-2, which increases both NKG2D and DAP-10 expression on murine cells [19], whereas TGF-β, which is elevated in active human TB disease but declines with chemotherapy [43], [44], down-regulates NKG2D expression on human CD8^+^ T and NK cells [45], [46]. Thus the altered cytokine milieu in active TB and its modulation with treatment will affect NKG2D expression.

NKG2D has multiple ligands, which are upregulated on stressed cells or infected cells in a complex manner [47]. Infection of monocytes and alveolar macrophages with *M. tuberculosis* leads to up-regulation of the ULBP1 ligand, and the NKG2D-ULBP1 interaction mediates NK cell-mediated killing [48]. Interestingly, cytomegalovirus co-infection can inhibit NKG2D ligand expression, leading to immune-suppression [49]. Furthermore, binding of soluble ligands, shed from activated CD4^+^ T cells, can lead to down-regulation of NKG2D expression and immune-suppression [50] and this mechanism may have been involved in the dramatic down-regulation of NKG2D observed in the intensive therapy phase of this study. Further studies in larger and diverse ethnic populations should be performed to validate these findings if NKG2D is to be useful as a biomarker for TB drug discovery. Changes in NKG2D in response to TB-treatment in HIV-positive people should also be determined. It would be particularly interesting to extend the functional characterization of CD8^+^NKG2D^+^ T cells during TB treatment, to determine their cytokine production and differentiation state.

Host biomarkers of TB treatment-response would revolutionize clinical trials of new drugs, by allowing sensitive measurement of drug efficacy early after treatment initiation and without reliance on microbiological assays or a 2-year relapse rate [6]. We have shown here that NKG2D expression is greatly reduced in peripheral blood during the intensive phase of successful TB treatment. Thus changes in peripheral NKG2D expression might be a useful contribution to a TB drug-efficacy biosignature in conjunction with other gene expression [40], secreted immune mediators and clinical parameters of treatment response. To be useful in TB drug development, potentially as a surrogate endpoint for the 2-year relapse rate in clinical trials, a TB drug-efficacy biosignature would need to be tested and validated in surrogate marker clinical trials, possibly alongside drug candidate clinical trials to improve feasibility and reduce cost. Ultimately, a TB drug-efficacy surrogate marker biosignature would dramatically reduce the cost of TB drug and regimen clinical trials, allowing earlier decision making and potentially the licensing of more compounds.
